# Polyphosphorhydrazone-Based Radical Dendrimers

**DOI:** 10.3390/molecules26051230

**Published:** 2021-02-25

**Authors:** Vega Lloveras, José Vidal-Gancedo

**Affiliations:** Institut de Ciència de Materials de Barcelona (ICMAB-CSIC) and CIBER-BBN, Campus UAB, 08193 Bellaterra, Spain

**Keywords:** radical dendrimers, organic radicals, PPH dendrimers, magnetic resonance imaging, contrast agents

## Abstract

The search for new biomedical applications of dendrimers has promoted the synthesis of new radical-based molecules. Specifically, obtaining radical dendrimers has opened the door to their use in various fields such as magnetic resonance imaging, as anti-tumor or antioxidant agents, or the possibility of developing new types of devices based on the paramagnetic properties of organic radicals. Herein, we present a mini review of radical dendrimers based on polyphosphorhydrazone, a new type of macromolecule with which, thanks to their versatility, new metal-free contrast agents are being obtained, among other possible applications.

## 1. Introduction

The search for new biomedical applications of dendrimers has promoted the synthesis of new radical-based molecules. Specifically, obtaining radical dendrimers (dendrimers functionalized with organic radicals) has opened the door to their use in various fields such as magnetic resonance imaging (MRI), as anti-tumor or antioxidant agents, or the possibility of developing new types of devices based on the paramagnetic properties of organic radicals. In particular, in MRI applications, paramagnetic gadolinium-based contrast agents (CAs) are widely used because, thanks to their seven unpaired electrons (spin 7/2), they shorten T_1_ relaxation time and lead to high signal enhancement [1,2,3,4,5]. Gadolinium dendrimeric systems have been used with success: for example, the covalent attachment of Gd(III) complexes to poly(amido amine) (PAMAM) dendrimers to generate unique macromolecular contrast agents for MRI has been reported by several research groups [6]. Although gadolinium-based contrast agents have historically been considered as safe, a linear relationship between their administration and the development of nephrogenic systemic fibrosis has been recognized in patients with renal impairment [7,8]. In addition to this, the accumulation of toxic Gd(III) ions in the brain, kidneys, liver, bones, or skin of patients with normal renal function has recently been demonstrated [9]. For this reason, it is crucial to find alternative imaging probes with the same or better paramagnetic properties than current Gd-based CAs.

More than 20 years ago, Janssen and Meijer [10] and Kashiwagi [11] functionalized dendrimers with organic radicals to study their electron paramagnetic resonance (EPR) or voltammetric behavior. Since then, only a few papers based mainly on poly(amido amine) (PAMAM) and poly(propylene imine) (PPI) dendrimers functionalized with organic radicals have been reported, with the majority of them devoted to the study of their magnetic or structural properties, relaxivity, or other related properties [12,13,14,15,16]. It is only a few years ago when, with a view to their application as contrast agents for MRI, they began to be studied in the scientific community [17,18,19,20,21,22,23,24]. Poly(propylenene imine), polyphosphorhydrazone (PPH), and oligoethylene glycol (OEG) based-dendrimers or dendrimer-like structures were functionalized with organic radicals, trying to replace the gadolinium III complexes currently used for this purpose. In fact, the first time the term “radical dendrimers” was used was in one of our first publications, specifically in the article published in *Macromolecules* in 2014, in which the synthesis and characterization of a family of polyphosphorhydrazone-based dendrimers fully functionalized with organic radicals was described for the first time [19].

Recently, radical dendrimers have also been used for their antioxidant or anti-tumor properties in TEMPO radical-terminated polyurethane dendrimers. In particular, the G4 generation exhibited significant anti-tumor activity and also showed a protective effect against oxidative cell damage [25,26].

## 2. Polyphosphorhydrazone-Based Radical Dendrimers

Focusing on polyphosphorhydrazone dendrimers, the synthesis of radical dendrimers based on PPH can only be understood thanks to the impressive work carried out by the group of Jean-Pierre Majoral and Anne-Marie Caminade using these phosphorous dendrimers [27,28,29,30,31,32].

**Figure 1:**
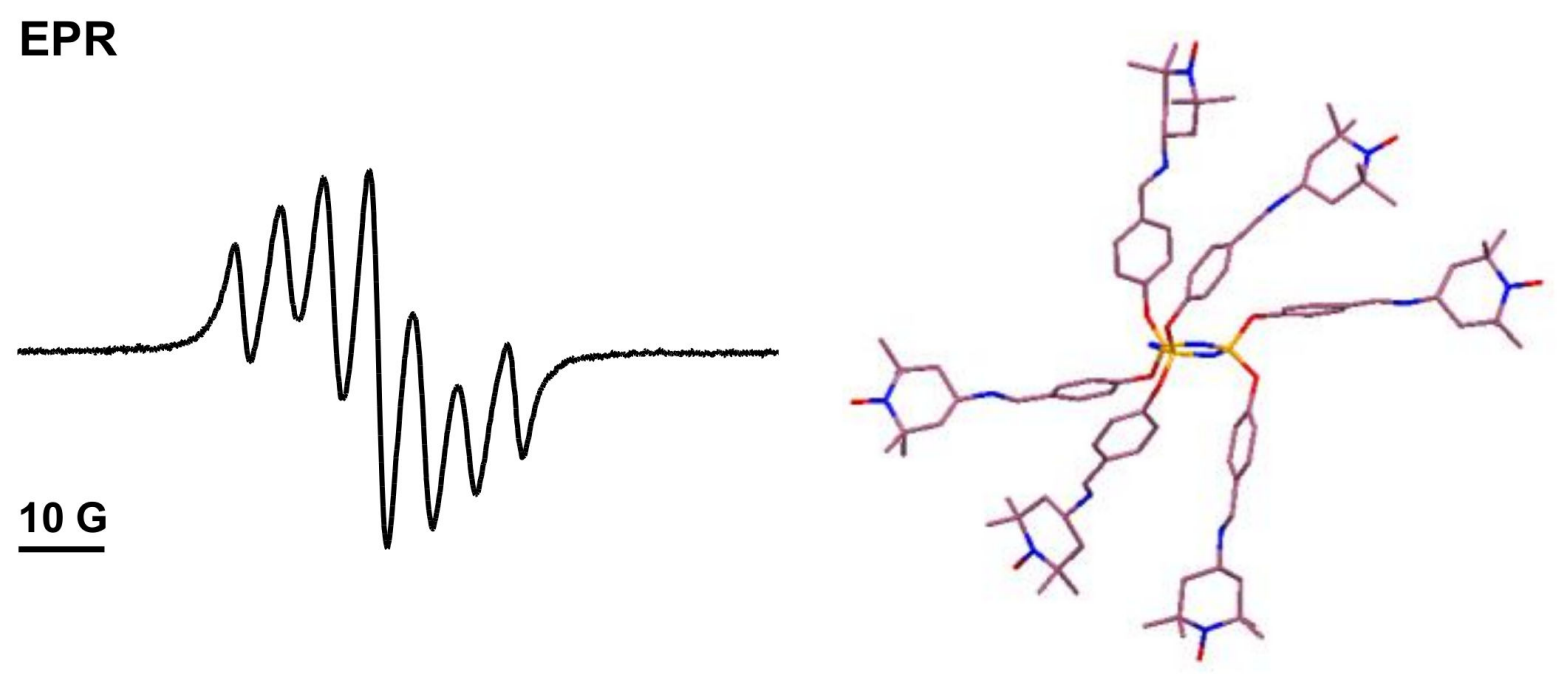
EPR spectrum at 350 K and the corresponding X-ray diffraction structure of cyclotriphosphazene functionalized with six TEMPO radicals. Published by *Org. Lett*. in 2013 [18].

The first articles published of this kind of dendrimer with radicals were based only on a cyclotriphosphazene ring as a scaffold for the radicals in order to study its magnetic, electrochemical, and structural properties, the interaction between radicals at the dendrimer surface, as well as the obtaining of chiral multi-spin systems [18,33,34,35,36,37,38]. For example, it was possible to provide, for the first time, experimental data about the molecular structure in solution, by EPR, of the cyclotriphosphazene ring functionalized with TEMPO radicals in its six branches, demonstrating they have the same relative orientation as in the solid state determined by X-ray diffraction: three branches above the cyclotriphosphazene ring and the other three below it (Figure 1) [18]. With a view to examining the possibility of building chiral multi-spin systems on the cyclotriphosphazene scaffold, Tamura and co-workers prepared chiral nitroxide–cyclotriphosphazene hybrid compounds, and they evaluated the magnitude of the intramolecular spin–exchange interactions by EPR, and their magnetic properties in the solid state by magnetic susceptibility measurements and X-ray crystallographic analysis [33,34].

**Scheme 1:**
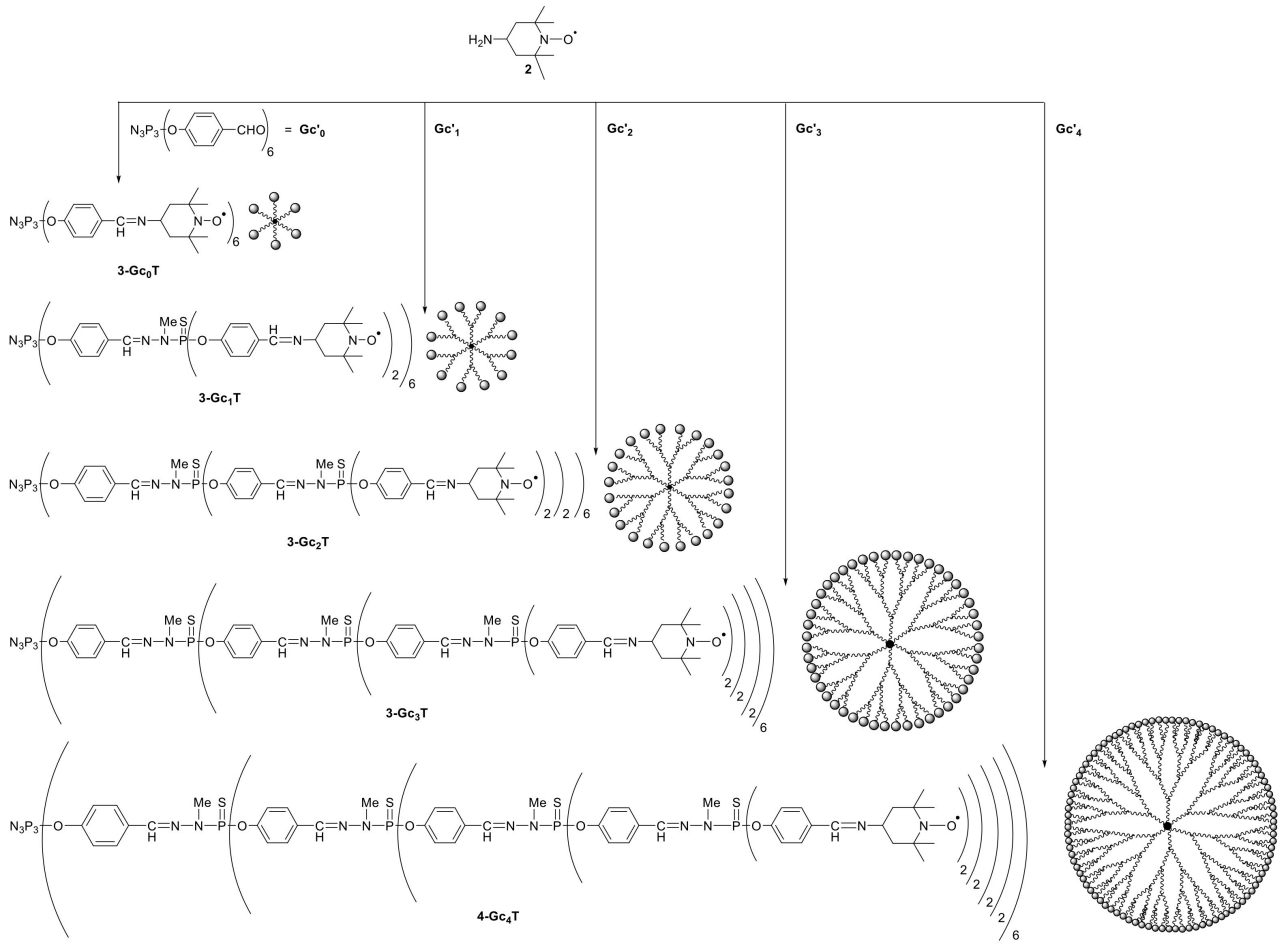
Synthesis of five generations of PPH-based radical dendrimers (3-Gc_0_T, 3-Gc_1_T, 3-Gc_2_T, 3-Gc_3_T, and 3-Gc_4_T), with 6, 12, 24, 48, and 96 TEMPO radical units, respectively, at the periphery. Published by *Macromolecules* in 2014 [19].

The first family of polyphosphorhydrazone dendrimers functionalized with organic radicals was published in 2014, as mentioned above. Vidal-Gancedo and co-workers obtained a five-generation family of PPH-based radical dendrimers with TEMPO as the radical end groups (Scheme 1) [19]. A comprehensive characterization was carried out by EPR, SQUID, ^1^H-NMR, ^31^P-NMR, FT-IR, and UV-Vis spectroscopy. The nitroxyl radicals at the periphery exhibited a strong intramolecular spin–exchange interaction among them, which depended on the dendrimer generation and the temperature. At higher generation and temperature, the intramolecular spin–exchange interaction among radicals was much stronger. The dynamic behavior with temperature of such an interaction was also carefully studied by EPR. In addition, the magnetic properties studied by SQUID magnetometry showed weak anti-ferromagnetic interaction between the spin carriers.

**Figure 2:**
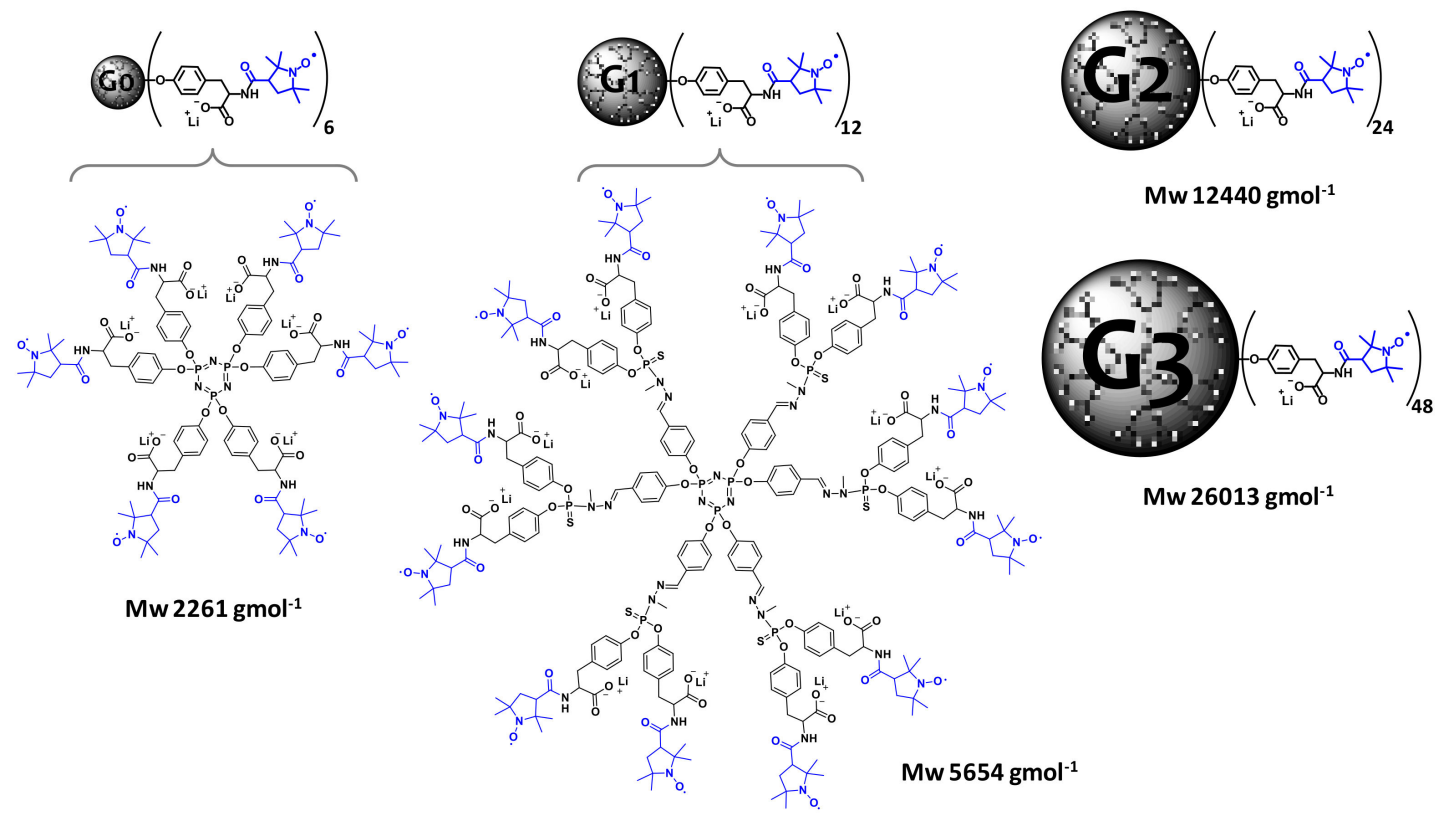
Structures of four generations of PPH Gn-Tyr-PROXYL (n = 0–3) radical dendrimers. Published by *ACS Appl. Bio Mater.* 2020 [21].

Having solved the challenge of obtaining PPH radical dendrimers, it is essential to solve one of the main problems that dendrimeric systems present, especially in middle and high generations: their solubility in water, if we are thinking about biomedical applications. The water solubility of dendrimers or similar compounds is sometimes overcome by PEGylation or by including free polar end groups, among other techniques [17,22,39,40]. However, in this way, not all of the anchoring positions at the end of the branches are available for radical anchoring, and hence the molecular relaxivity should be lower than with a fully nitroxide covered surface. In our group, we proposed an innovative procedure to increase the water solubility and, at the same time, to maintain the entire number of terminal functional groups to be functionalized with radicals. It consists of the use of an amino acid, in this case tyrosine (Tyr), as a linker between the PPH branches and the radicals, in this case PROXYL radicals (Gn-Tyr-PROXYL dendrimers, Figure 2) [21]. The amino acid is coupled to the dendrimer’s branches, providing an available amino group for radical coupling and a methyl ester group to provide negative charges via hydrolysis, which confer high solubility at physiological pH and low toxicity for intravenous administration. In this way, we obtained a series of radical dendrimers fully soluble in water with up to 48 PROXYL radicals in the periphery that provided very high relaxivities.

**Figure 3:**
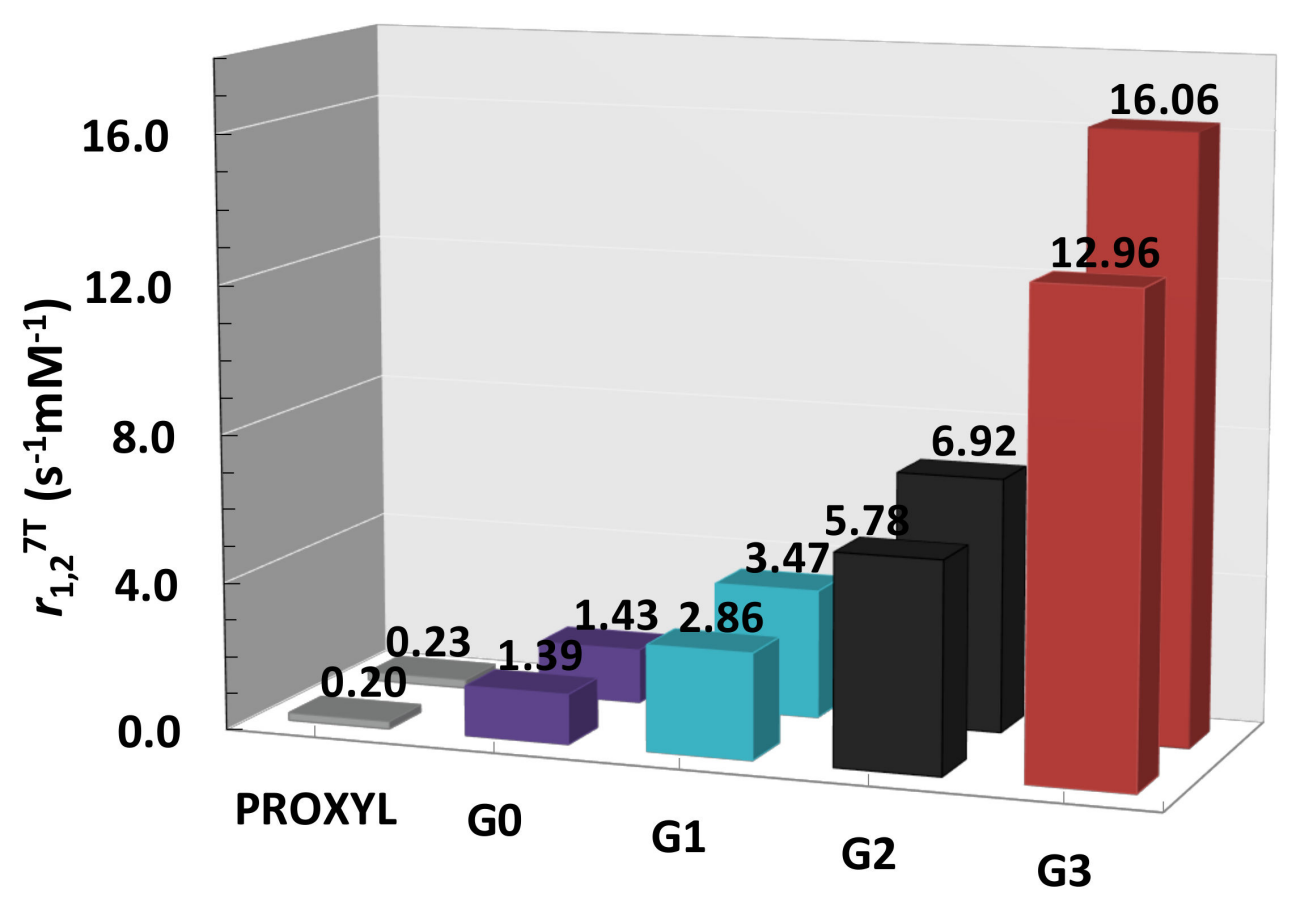
Longitudinal (r_1_) and transverse (r_2_) relaxivity per molecule of PPH Gn-Tyr-PROXYL (n = 0,3) radical dendrimers determined at 7T in 100 mM phosphate buffer pH 7.4, 300 K. Published by *ACS Appl. Bio Mater*. in 2020 [21].

The fully water-soluble PPH based Gn-Tyr-PROXYL radical dendrimers obtained offered to the pendant radicals much higher stability against reduction compared with the free PROXYL radical, as shown by an EPR chemical reduction study with ascorbic acid. The high number of paramagnetic nitroxide units in the dendrimer’s periphery resulted in high molecular relaxivities (Figure 3): while G1-Tyr-PROXYL dendrimer presented a similar relaxivity (2.9 mM^−1^s^−1^) than the most widely used Gd-based CA in clinics, Magnevist (Gd-DTPA, 3.2 mM^−1^s^−1^ r.t. 7T), one molecule of G3-Tyr-PROXYL radical dendrimer presented a relaxivity of ca. 13 mM^−1^s^−1^ that means a remarkably 4 times higher value than Gd-DTPA. Similar results were obtained in the ex-vivo MRI studies in mice, obtaining very good relative contrast enhancement after the local injection of G3-Tyr-PROXYL radical dendrimer compared with Gd-DTPA. Moreover, the four radical dendrimers’ generations showed very low cytotoxicity. These studies all demonstrated that such radical dendrimers are excellent candidates to be used as MRI contrast agents suited for biomedical applications. Furthermore, the control over the structure of these dendrimers, opens the window of opportunity for a future modulation of their biodistribution profiles.

It is important to note that the use of radical dendrimers allows us to tune the system by playing with the type of dendrimer, its branches, the radical used, and the linker to connect the radical to the dendrimer. In this way, we can modify the properties of the radical dendrimers at will, opening the perspective of creating a system with optimized properties to fulfill the requests of each application.

There are several examples that allow us to see the versatility of this approach: one of them is the improvement of the solubility in water explained in the previous example, using amino acids as linkers [21].

**Figure 4:**
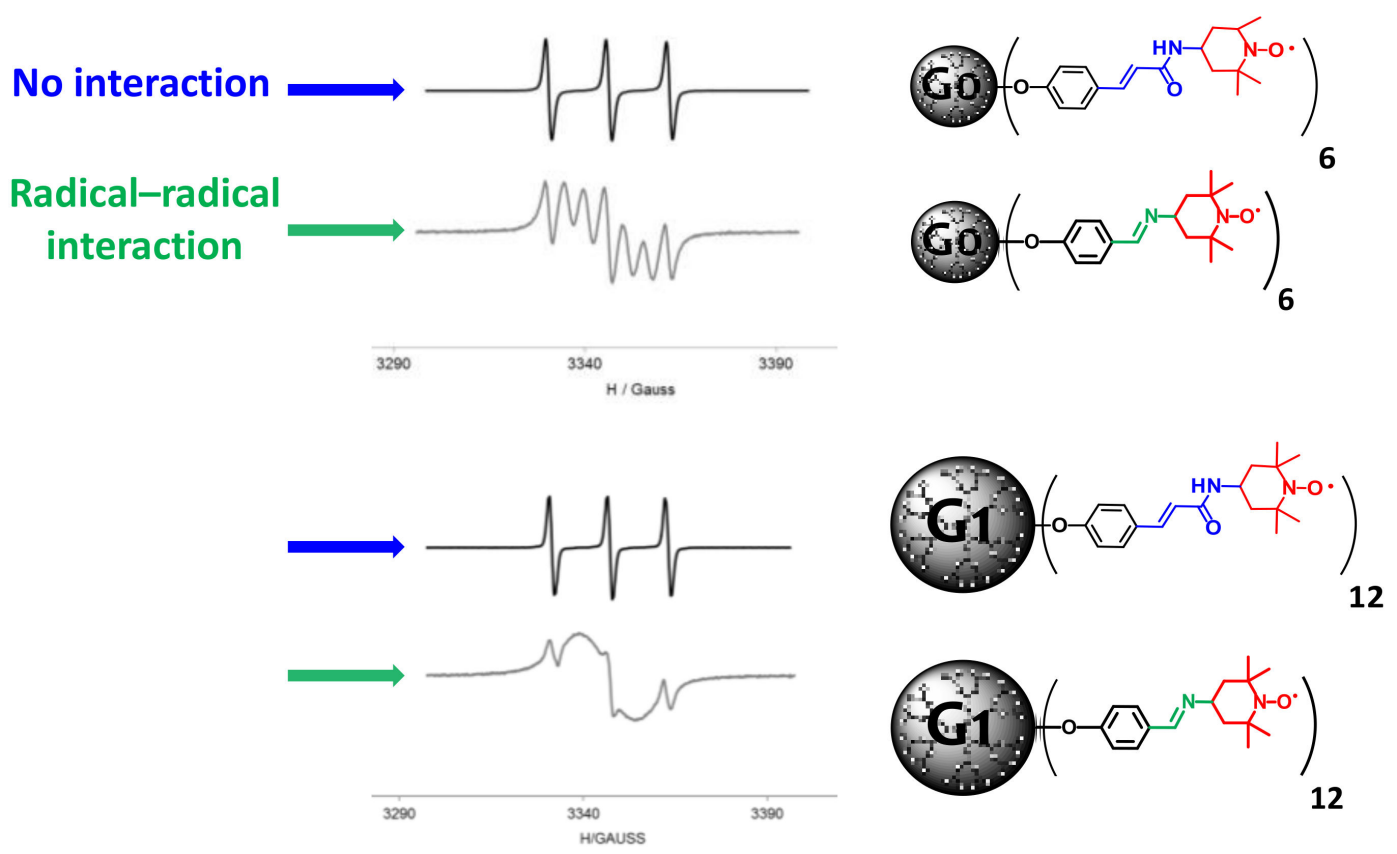
EPR spectra of PPH Gn-acrylamido-TEMPO and Gn-imino-TEMPO radical dendrimers, showing the different intramolecular spin–exchange interaction among radicals, depending on the linker used. Published by *ChemPhysChem* in 2018 [41].

Another example, is the possibility to tune the intramolecular spin–exchange interaction between radicals, as well as their physical–chemical properties, by using different linkers. Two generations of PPH-based radical dendrimers were synthesized with two different linkers, imino and acrylamido ones (Figure 4) [41]. In this paper, a drastic change in the way that the radicals interacted among one another was observed by EPR and CV studies. While the radicals in Gn-imino-TEMPO dendrimers presented a strong intramolecular spin–exchange interaction, in the Gn-acrylamido-TEMPO ones, they acted mainly as independent radicals. In addition, the choice of the linker can lead to a radical dendrimer with different physical–chemical properties, even though the dendritic structure is the same—for instance, showing completely different polarity (in this case, much higher with the acrylamido linker than with the imino one). The control over the interactions between radicals opens the perspective to tune them as needed in many applications, such as dynamic nuclear polarization (DNP), MRI, organic electronics, or organic batteries.

**Figure 5:**
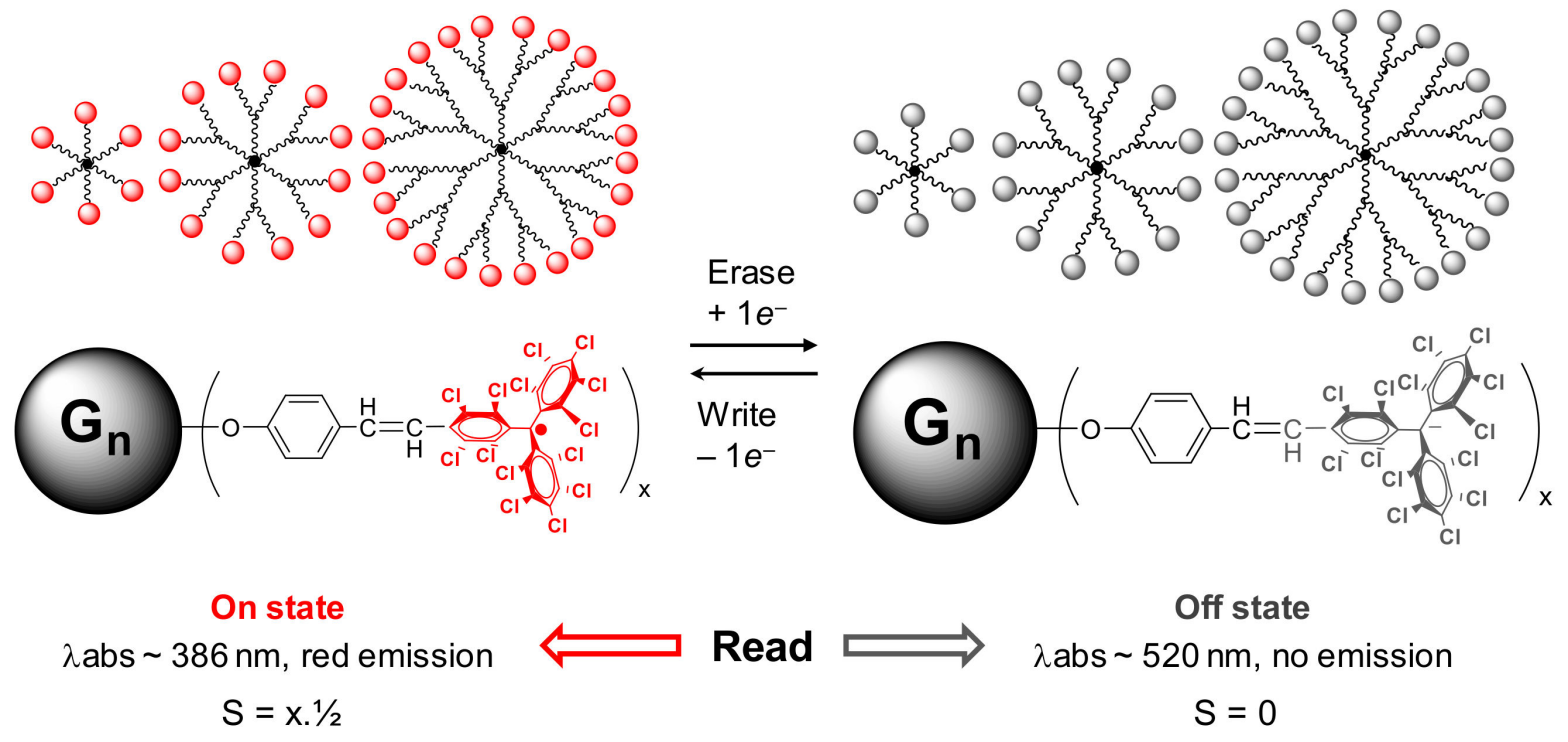
Representation of the electrochemical bistability of PTM radical dendrimers (PPH Gn(PTM^•^)_x_, n = 0–2, x = 6–24). The PTM molecule has two redox states, the radical (left) and anion (right) forms, which can be electrochemically interconverted. Each state exhibits different magnetic and optical properties: radical dendrimers Gn(PTM^•^)_x_ exhibit spin S = x·½, an absorbance band at 386 nm, and red fluorescence emission, and anion Gn(PTM^−^)_x_ is a diamagnetic species with S = 0, a maximum absorbance band at ca. 520 nm, and is not fluorescent. Published by *Chem. Mater.* In 2019 [42].

Finally, if we change the type of radical—for instance, using perchlorotriphenylmethyl (PTM) radicals—we can take advantage of their redox properties, for example in the design of different devices such as molecular switches [42]. Three generations of polyphosphorhydrazone dendrimers, fully functionalized with 6, 12, and 24 PTM radicals in the periphery, were capable of undergoing electrochemical reversible switching by multi-electron reduction and oxidation. An electrical input was used to trigger the physical properties of these radical dendrimers in a reversible way, modifying their optical, magnetic, and electronic properties (Figure 5).

## 3. Relaxivity Values Obtained So Far with Radical Dendrimers

As one of the main applications of radical dendrimers described to date is as contrast agents for magnetic resonance imaging, herein, we present a short overview of the relaxivity results obtained so far for the different radical dendrimers soluble in water synthesized for such an application. Contrast agents are divided into two principal classes: T_1_ contrast agents (e.g., paramagnetic metals such as gadolinium) that give positive-contrast images by locally reducing the water ^1^H longitudinal relaxation time (T_1_), and T_2_ contrast agents (e.g., superparamagnetic iron oxide nanoparticles) that lead to negative-contrast images by locally decreasing the water ^1^H transverse relaxation time (T_2_). The corresponding water ^1^H relaxivities of a contrast agent (r_1_ and r_2_, respectively) describe the extent to which the agent decreases the T_1_ and T_2_ times of water ^1^H in the presence of 1 mM concentration of the agent. Higher relaxivity values translate to a better image contrast and, hence, better MRI efficiency of the system. As explained in the introduction, paramagnetic systems such as Gd-based contrast agents or organic radical-based systems such as radical dendrimers act mainly as effective T_1_ contrast agents.

When PAMAM and PPI (DAB-type) dendrimers were fully functionalized with up to 32 PROXYL radicals per macromolecule, the relaxivities obtained significantly increased, up to r_1_ ca. 5 s^−1^mM^−1^ at 1.5 T, compared to those of the monomeric nitroxide with a single spin (0.18 s^−1^mM^−1^). However, in the highest generation, the relaxivity per nitroxide unit was lower than the free radical (0.16 s^−1^mM^−1^) [12]. When PAMAM dendrimers (G0-NH_2_ to G4-NH_2_) were fully functionalized with nitronyl nitroxide (NIT) radicals, the relaxivity per radical unit obtained in G2-NIT and G3-NIT was about half of the value for the monomeric free radical in solution, and the authors attributed this fact to the restricted volume, where water molecules could approach the paramagnetic center in dendrimers [15]. In these two previous reported works, the relaxivity per radical unit decreased in higher generations, which can be explained by the aggregation problems promoted by the lower water solubility of larger generations, what makes radical units inaccessible by water molecules. The water solubility problems were overcome in different ways. Using an aminoacid as a linker between the radical and the dendrimer’s branch, Vidal-Gancedo and co-workers achieved fully water-soluble radical dendrimers with a relaxivity per molecule of r_1_ ca. 13 s^−1^mM^−1^ at 7 T in a PPH-G3 generation with 48 PROXYL radicals anchored (G3-Tyr-PROXYL), and an increase on a per radical unit value, from the free PROXYL (0.20 s^−1^mM^−1^) to PPH-G3 (0.27 s^−1^mM^−1^) [21]. The same authors used OEG-based dendrimers, i.e., dendrimers with water-soluble branches themselves, to anchor PROXYL radicals, also obtaining highly soluble radical dendrimers. They achieved a relaxivity per molecule of r_1_ 3.4 s^−1^mM^−1^ at 7 T with the second generation (20 radicals units), and a similar r_1_ per nitroxide unit than the free radical [20]. Rajca and co-workers introduced poly(ethylene glycol) chains into spirocyclohexyl nitroxide-based PPI dendrimers, resulting in a relaxivity per molecule of r_1_ ca. 5 s^−1^mM^−1^ at 7 T in G4-PP-PEG system with an average number of nitroxide units per macromolecule of 13, which means a relaxivity per nitroxide unit of 0.42 s^−1^mM^−1^ [17]. The authors pointed out high rotational correlation times of the macromolecule (restricted motion of the radicals) and long residence/exchange time of water molecules hydrogen-bonded to the nitroxide radical as the most probable factors limiting the water r_1_. Other related structures have also been studied as MRI contrast agents, such as brush-arm star polymer or cross-linked polymers with nitroxides anchored, reporting very high r_1_ and even r_2_ per nitroxide unit [22,39]; however, as they are not radical dendrimers, they are beyond the scope of this review.

## 4. Conclusions

Dendrimers offer many applications due to their functional and structural versatility. In fact, radical dendrimers (i.e., dendrimers functionalized with persistent organic radicals) offer us the possibility to play with different types of dendrimers or radicals, as well as with the linker between them modulating their properties to fulfill the requests of each application.

In particular, radical dendrimers based on PPH offer many possibilities. For example, as contrast agents in magnetic resonance imaging (MRI), in order to replace gadolinium-based contrast agents, thanks to their high relaxivity and low toxicity, or the possibility of developing new types of devices based on the paramagnetic properties of organic radicals. Other applications that have begun to be developed in other types of radical dendrimers, such as anti-tumor or antioxidant properties, can surely also be studied in PPH-based radical dendrimers. In short, the development of this type of new compounds opens up a wide range of possibilities in the future, mainly in biomedical applications.

## References

[1] Swanson S.D., Kukowska-Latallo J.F., Patri A.K., Chen C., Ge S., Cao Z., Kotlyar A., East A.T., Baker J.R. (2008). Targeted gadolinium-loaded dendrimer nanoparticles for tumor-specific magnetic resonance contrast enhancement. Int. J. Nanomed..

[2] Caravan P., Ellison J.J., McMurry T.J., Lauffer R.B. (1999). Gadolinium(III) Chelates as MRI Contrast Agents: Structure, Dynamics, and Applications. Chem. Rev..

[3] Wahsner J., Gale E., Rodríguez-Rodríguez A., Caravan P. (2019). Chemistry of MRI contrast agents: Current challenges and new frontiers. Chem. Rev..

[4] Cao Y., Xu L., Kuang Y., Xiong D., Pei R. (2017). Gadolinium-based nanoscale MRI contrast agents for tumor imaging. J. Mater. Chem. B.

[5] McMahon M.T., Bulte J.W.M. (2018). Two decades of dendrimers as versatile MRI agents: A tale with and without metals. Wiley Interdiscip. Rev. Nanomed. Nanobiotechnol..

[6] Kojima C., Turkbey B., Ogawa M., Bernardo M., Regino C.A.S., Bryant L.H., Choyke P.L., Kono K., Kobayashi H. (2011). Dendrimer-based MRI contrast agents: The effects of PEGylation on relaxivity and pharmacokinetics. Nanomed. Nanotechnol. Biol. Med..

[7] Rogosnitzky M., Branch S. (2016). Gadolinium-based contrast agent toxicity: A review of known and proposed mechanisms. BioMetals.

[8] Ranga A., Agarwal Y., Garg K. (2017). Gadolinium based contrast agents in current practice: Risks of accumulation and toxicity in patients with normal renal function. Indian J. Radiol. Imaging.

[9] Olchowy C., Cebulski K., Łasecki M., Chaber R., Olchowy A., Kałwak K., Zaleska-Dorobisz U. (2017). The presence of the gadolinium-based contrast agent depositions in the brain and symptoms of gadolinium neurotoxicity—A systematic review. PLoS ONE.

[10] Bosman A.W., Janssen R.A.J., Meijer E.W. (1997). Five Generations of Nitroxyl-Functionalized Dendrimers. Macromolecules.

[11] Kashiwagi Y., Kurashima F., Kikuchi C., Anzai J.-I., Osa T. (1999). Voltammetric behavior of poly(amidoamine) dendrimers containing nitroxyl radical end groups. Electrochem. Commun..

[12] Winalski C.S., Shortkroff S., Mulkern R.V., Schneider E., Rosen G.M. (2002). Magnetic resonance relaxivity of dendrimer-linked nitroxides. Magn. Reson. Med..

[13] Maliakal A.J., Turro N.J., Bosman A.W., Cornel J., Meijer E.W. (2003). Relaxivity studies on dinitroxide and polynitroxyl functionalized dendrimers: Effect of electron exchange and structure on paramagnetic relaxation enhancement. J. Phys. Chem. A.

[14] Yordanov A.T., Yamada K., Krishna M.C., Mitchell J.B., Woller E., Cloninger M., Brechbiel M.W. (2001). Spin-Labeled Dendrimers in EPR Imaging with Low Molecular Weight Nitroxides. Angew. Chem. Int. Ed..

[15] Francese G., Dunand F.A., Loosli C., Merbach A.E., Decurtins S. (2003). Functionalization of PAMAM dendrimers with nitronyl nitroxide radicals as models for the outer-sphere relaxation in dentritic potential MRI contrast agents. Magn. Reson. Chem..

[16] Sebby K.B., Walter E.D., Usselman R.J., Cloninger M.J., Singel D.J. (2011). End-Group Distributions of Multiple Generations of Spin-Labeled PAMAM Dendrimers. J. Phys. Chem. B.

[17] Rajca A., Wang Y., Boska M., Paletta J.T., Olankitwanit A., Swanson M.A., Mitchell D.G., Eaton S.S., Eaton G.R., Rajca S. (2012). Organic Radical Contrast Agents for Magnetic Resonance Imaging. J. Am. Chem. Soc..

[18] Badetti E., Lloveras V., Wurst K., Sebastián R.M., Caminade A.-M., Majoral J.-P., Veciana J., Vidal-Gancedo J. (2013). Synthesis and structural characterization of a dendrimer model compound based on a cyclotriphosphazene core with TEMPO radicals as substituents. Org. Lett..

[19] Badetti E., Lloveras V., Muñoz-Gómez J.L., Sebastián R.M., Caminade A.M., Majoral J.P., Veciana J., Vidal-Gancedo J. (2014). Radical dendrimers: A family of five generations of phosphorus dendrimers functionalized with TEMPO radicals. Macromolecules.

[20] Zhang S., Lloveras V., Pulido D., Liko F., Pinto L.F., Albericio F., Royo M., Vidal-Gancedo J. (2020). Radical Dendrimers Based on Biocompatible Oligoethylene Glycol Dendrimers as Contrast Agents for MRI. Pharmaceutics.

[21] Pinto L.F., Lloveras V., Zhang S., Liko F., Veciana J., Muñoz-Gómez J.L., Vidal-Gancedo J. (2020). Fully Water-Soluble Polyphosphorhydrazone-Based Radical Dendrimers Functionalized with Tyr-PROXYL Radicals as Metal-Free MRI T 1 Contrast Agents. ACS Appl. Bio Mater..

[22] Guo S., Wang X., Dai Y., Dai X., Li Z., Luo Q., Zheng X., Gu Z., Zhang H., Gong Q. (2020). Enhancing the Efficacy of Metal-Free MRI Contrast Agents via Conjugating Nitroxides onto PEGylated Cross-Linked Poly(Carboxylate Ester). Adv. Sci..

[23] Li H., Sun J., Zhu H., Wu H., Zhang H., Gu Z., Luo K. (2020). Recent advances in development of dendriticpolymer-based nanomedicines for cancer diagnosis. Wiley Interdiscip. Rev. WIREs Nanomed. Nanobiotechnol..

[24] Chis A.A., Dobrea C., Morgovan C., Arseniu A.M., Rus L.L., Butuca A., Juncan A.M., Totan M., Vonica-Tincu A.L., Cormos G. (2020). Applications and Limitations of Dendrimers in Biomedicine. Molecules.

[25] Ali B.M., Boothapandi M., Sultan Nasar A. (2020). Nitric oxide, DPPH and hydrogen peroxide radical scavenging activity of TEMPO terminated polyurethane dendrimers: Data supporting antioxidant activity of radical dendrimers. Data Brief.

[26] Ali B.M., Velavan B., Sudhandiran G., Sridevi J., Sultan Nasar A. (2020). Radical dendrimers: Synthesis, anti-tumor activity and enhanced cytoprotective performance of TEMPO free radical functionalized polyurethane dendrimers. Eur. Polym. J..

[27] Launay N., Caminade A.-M., Majoral J.P. (1997). Synthesis of bowl-shaped dendrimers from generation 1 to generation 8. J. Organomet. Chem..

[28] Launay N., Caminade A.-M., Majoral J.-P. (1995). Synthesis and Reactivity of Unusual Phosphorus Dendrimers. A Useful Divergent Growth Approach Up to the Seventh Generation. J. Am. Chem. Soc..

[29] Blais J.-C., Turrin C.-O., Caminade A.-M., Majoral J.-P. (2000). MALDI TOF Mass Spectrometry for the Characterization of Phosphorus-Containing Dendrimers. Scope and Limitations. Anal. Chem..

[30] Majoral J.P., Caminade A.M. (1999). Dendrimers containing heteroatoms (si, p, B, ge, or bi). Chem. Rev..

[31] Caminade A.-M., Ouali A., Laurent R., Turrin C.-O., Majoral J.-P. (2015). The dendritic effect illustrated with phosphorus dendrimers. Chem. Soc. Rev..

[32] Caminade A.-M., Turrin C.-O., Majoral J.-P. (2018). Phosphorus Dendrimers in Biology and Nanomedicine.

[33] Shimono S., Tamura R., Ikuma N., Takahashi H., Sakai N., Yamauchi J. (2004). Characterization of the Chiral Paramagnetic Multispin System Built on a Cyclotriphosphazene Scaffold. Chem. Lett..

[34] Shimono S., Takahashi H., Sakai N., Tamura R., Ikuma N., Yamauchi J. (2005). Use of Cyclotriphosphazene as a Molecular Scaffold for Building Chiral Multispin Systems. Mol. Cryst. Liq. Cryst..

[35] Fidan I., Önal E., Yerli Y., Luneau D., Ahsen V., Hirel C. (2017). Synthetic Access to a Pure Polyradical Architecture: Nucleophilic Insertion of Nitronyl Nitroxide on a Cyclotriphosphazene Scaffold. Chempluschem.

[36] Fidan I., Önal E., Yerli Y., Luneau D., Ahsen V., Hirel C. (2016). Synthesis and Straightforward Quantification Methods of Imino Nitroxide-Based Hexaradical Architecture on a Cyclotriphosphazene Scaffold. Inorg. Chem..

[37] Lloveras V., Badetti E., Wurst K., Vidal-Gancedo J. (2015). Synthesis, X-Ray Structure, Magnetic Properties, and a Study of Intra/Intermolecular Radical-Radical Interactions of a Triradical TEMPO Compound. ChemPhysChem.

[38] Dulog L., Mohler H. (1988). A λ^5^-cylotriphosphazene with nitroxyl groups as model for a paramagnetic poly(organo-λ^5^-phosphazene. Makromol. Chem..

[39] Nguyen H.V.T., Chen Q., Paletta J.T., Harvey P., Jiang Y., Zhang H., Boska M.D., Ottaviani M.F., Jasanoff A., Rajca A. (2017). Nitroxide-Based Macromolecular Contrast Agents with Unprecedented Transverse Relaxivity and Stability for Magnetic Resonance Imaging of Tumors. ACS Cent. Sci..

[40] Niidome T., Gokuden R., Watanabe K., Mori T., Naganuma T., Utsumi H., Ichikawa K., Katayama Y. (2014). Nitroxyl radicals-modified dendritic poly(l-lysine) as a contrast agent for Overhauser-enhanced MRI. J. Biomater. Sci. Polym. Ed..

[41] Lloveras V., Liko F., Pinto L.F., Muñoz-Gómez J.L., Veciana J., Vidal-Gancedo J. (2018). Tuning Spin-Spin Interactions in Radical Dendrimers. ChemPhysChem.

[42] Lloveras V., Liko F., Muñoz-Gómez J.L., Veciana J., Vidal-Gancedo J. (2019). Redox-Active PTM Radical Dendrimers as Promising Multifunctional Molecular Switches. Chem. Mater..

